# Effectiveness of Extracorporeal Shock Wave Therapy Reduces Leg Cramps in Patients of Lumbar Degenerative Disorders: A Retrospective Study

**DOI:** 10.1155/2021/3554397

**Published:** 2021-10-25

**Authors:** Bang-zhi Li, Heng-fei Li, Zhi-wen Zhang, Yang Li, Wei Wang, Yong Xiong

**Affiliations:** ^1^College of Acupuncture and Orthopedics, Hubei University of Chinese Medicine, Wuhan, HuBei 430061, China; ^2^Hubei Provincial Hospital of Traditional Chinese Medicine, Wuhan 430061, China

## Abstract

**Background:**

The extracorporeal shock wave therapy (ESWT) has been fully utilized in orthopedics, but there are few studies in the treatment of lower limb spasm and pain caused by lumbar degenerative disorders (LDD). This study assesses the influence of ESWT in patients with LDD.

**Methods:**

From October 2017 to June 2019, 126 patients with LDD were enrolled. All patients received shock wave therapy, once every two days for four weeks in total. Each treatment consisted of 2,000 shocks with a frequency of 8-10 shocks per second. To analyze the therapeutic progress, the following tests were performed (before and after therapy; 1- and 3-month follow-up) to assess pain and functional efficiency: (1) Visual Analog Scale (VAS), (2) the frequency and duration of muscle cramps, and (3) Fugl-Meyer (LL).

**Results:**

Mean BMI of the participants was 26.1 ± 3.0 kg/m^2^. There was no statistically significant difference in terms of age or BMI between the groups (*p* > 0.05). Although all scoring parameters improved in both groups, the improvement in the ESWT group was more pronounced in pain (*p* < 0.001 and *p* < 0.001, respectively). A review of the LMA scores of our patients demonstrated moderate functional limitations before treatment and increased functional status after treatment in all patients, while overall functional status was fully improved in patients of the ESWT group (*p* < 0.001).

**Conclusion:**

The ESWT is particularly effective effect for patients with LDD. The use of ESWT has a significant long-term influence on the reduction of pain, leg cramps, and the improvement of the general functional state in relation to the conventional motor improvement program.

## 1. Introduction

Muscle cramp is fundamentally a medical problem but also a sociological and an economic one. Muscle cramps which characterized by the painful, involuntary, and paroxysmal contraction of a muscle are common and can occur in a wide range of settings and are a highly distressing condition. The symptoms lead to a decrease in the quality of life for patients and sometimes disturb the proper functioning of the entire body, stimulating the development of several complications and comorbidities. Under certain physiological conditions, cramps are prone to occur, such as older adults, pregnant women, and athletes can cramp when they are pushing the limits of their endurance [1–5]. Some people develop muscle cramps as a symptom of other medical conditions. A large number of studies have proved that one out of every three people in a healthy population has had at least one muscle cramp during the previous year [6, 7], which may include trunk and limbs. In the elderly, this number will be higher, and muscle cramps may occur even when resting [3]. At present, clinically, more attention is paid to the pain, function, and sensory disturbances caused by LDD, while the physical and mental damage caused by muscle spasm to LDD patients is ignored. The social benefits of relieving lower extremity muscle spasms have also been underestimated [8–11].

LDD may cause pain and functional and sensory disturbances in multiple parts. Conservative treatment is often used in the early stage. If conservative treatment fails, surgical treatment is recommended. After standard treatment, the symptoms will be alleviated in most cases. However, whether it is conservative treatment or surgical treatment, the side effects caused by lower extremity muscle spasm caused by nerve involvement are usually not significantly improved and may even develop further [9–11]. In LDD patients, lower limb spasms often occur at rest, especially at night, and severe pain can make it difficult for patients to fall asleep. The pathological mechanism that LDD patients are more prone to spasm may be due to the long-term interference of the spinal cord and nerves. In this study, some patients who have undergone decompression surgery are included. Some of them still suffer from lower limb cramps. 46% of them believe that there is no improvement in lower limb cramps before and after surgery, and 16% of the patient even thinks that cramps have worsened after the operation. Therefore, more and more spine surgeons now use the spasm induction test as an effective diagnostic tool for the diagnosis of LDD, such as instructing patients to resist resistance to tighten muscles and cause muscle spasms. A study by Demircan and colleagues [8] proved that a negative cramp induction experiment was positively correlated with postoperative satisfaction of LDD patients. Therefore, it is necessary to find a treatment that can effectively alleviate leg cramps in LDD patients.

Shock wave therapy is an effective method to dissolve calcification and stimulate tissue regeneration by generating high-energy mechanical impulses through regular alternating compression and decompression processes [12, 13]. In the 1970s, shock wave therapy was widely used to treat kidney and gallbladder stones. With the development of science and medicine in the past few years, more and more orthopedic professionals have reported the use of shock waves to treat various pain conditions and musculoskeletal disorders, including myofascial pain syndrome, knee joint pain, rotator cuff tendinitis, sacroiliac joint pain, tendinopathy, enthesopathy, and calcifications [14]. However, the mechanism of extracorporeal shock wave therapy (ESWT) is still not fully understood. Current studies have proved that shock wave therapy can promote tissue healing and calcification. At the same time, ESWT treatment is still lacking in the treatment of lumbar degenerative disorders [14, 15].

In this study, we aimed to investigate the efficacy of ESWT (ESWT) in relieving pain and improving leg cramps in the treatment of patients with LDD.

## 2. Materials and Methods

### 2.1. Inclusion and Exclusion Criteria

Each patient provided informed consent for participation in the study. This retrospective study was conducted in accordance with the Declaration of Helsinki (Ethical Principles for Medical Research Involving Human Subjects) and was approved by the Regional Ethics Board of Hubei Medical.

This study was designed as a retrospective study to explore the effect of ESWT on lower extremity muscle spasms in LDD. A total of 126 patients (51 male participants and 75 female participants) underwent ESWT curing from October 2017 to June 2019. The age of participants ranged from 34 to 84 years, with a mean of 62.27 ± 16.18 years.

Inclusion criteria for the study were as follows: patients who have been diagnosed with LDD and have symptoms of leg muscle cramps, including those who have undergone lumbar fusion surgery and still have symptoms of lower extremity muscle spasms and have undergone standardized ESWT in Hubei Provincial Hospital of Traditional Chinese Medicine.

Exclusion criteria for the study were as follows: (1) patients who have lost follow-up of treatment effect.

### 2.2. Treatment Method

Before treatment, the patient has been fully informed of the purpose of treatment and possible consequences, so as not to cause unnecessary trepidation and other psychological discomfort. ESWT group used ESWT treatment and conventional physical therapy, including Chinese herbal compress (30 min) and muscle pulse stimulation. The ESWT instrument is SPE-02 (DeJiang NV, Shenzhen, China). Set up the ESWT treatment instrument to the parameters (8 Hz/s, 2500 times) what is needed for treatment. Instruct the patient to lie down, and the surgeon evenly applies the ultrasound gel to the treatment area's skin (including the waist, back, and affected limbs). Use the probe to act on the erector spine muscle abdomen and the patient's muscle abdomen. The purpose of the ultrasound gel is to reduce tissue resistance and maintain energy transmission.

Before and after treatment, all patients were evaluated for age, height, weight, body mass index (BMI), and pain intensity assessed using a Visual Analog Scale; the frequency and duration of muscle cramps are recorded; lower limb function is assessed using a Fugl-Meyer. The motor function domain of FMA was used to assess UL and LL impairment. This domain has a total of 100 points for normal motor function. Maximum scores are 66/100 for UL and 34/100 for the LL section. The evaluation includes measurement of voluntary movement, velocity, coordination, and reflex activity, through an ordinal scale applied to each item: 0, cannot be performed; 1, partially performed; and 2, performed completely. According to the FMA-UL scores [16], motor impairment was classified as severe (less than 32 points), moderate (scores between 32 and 47), or mild (equal or more than 48 points) [17]. Participants were also classified according their LL motor impairment as severe (less than 19), moderate (scores between 20 and 28), or mild (equal or more than 29 points) [18]. This study only involves the lower extremities.

### 2.3. Statistical Analyses

The IBM SPSS version 22.0 software (IBM Corp., Armonk, NY, USA) was used for statistical analyses. This analysis revealed a standard effect size of 0.72 and at least 80 cases with a 95% confidence interval and a power of 80%. In order to assess the difference between the groups, the Mann–Whitney *U* test was used in continuous data which were independent from each other among the groups. The Wilcoxon test was used to assess the pre- and posttreatment variables. A *p* value lower than 0.05 was considered statistically significant.

## 3. Results

The average BMI of the participants was 26.1 ± 3.0 kg/m^2^. The sociodemographic variables are show in Table 1. There was no statistically significant differences in age or BMI between the groups (*p* > 0.05). Although there were significant improvements in all evaluation parameters in the two groups, patients in the ESWT group had statistically significant improvements in pain and cramp frequency and duration (*p* < 0.001 and *p* < 0.001, respectively) (Table 2). A review of the LMA scores of our patients demonstrated that all patients had functional limitations before treatment and improved functional status after treatment, while the overall functional status of patients in the ESWT group was fully improved (*p* < 0.001).

## 4. Discussion

In recent years, lumbar degenerative disease has gradually become one of the important public health problems that endanger human health and has become the most common type of disease in orthopedics. Its clinical symptoms are mainly low back pain, lower extremity paresthesia, and lower extremity muscle cramps. A muscle spasm is a neuromuscular phenomenon in which muscles contract involuntarily, accompanied by severe pain and dysfunction. Past studies have proved that ESWT can relieve pain and increase the function of the affected area. It is an effective treatment method in clinical practice and is widely used in various pain disorders, especially chronic injuries [19].

The prevalence age of LDD is between 45 and 70 years old. The average age of patients in this study was 62.24 ± 16.18 years. Previous researchers evaluated the relationship between BMI and LDD and found that obesity is an important risk factor for the development of LDD. The average BMI in our study was 27.1 ± 3.6 kg/m^2^. On the other hand, it may be due to female plays more important role in housework activities makes women more susceptible to LDD, younger age of onset, and more severe symptoms [20].

Regarding the treatment of LDD, it is generally accepted in clinical practice that early use of various drugs for improving circulation, pain relief, and neurotrophic nerves, combined with various physiotherapy and other conservative treatments, can relieve symptoms. At the same time, conservative methods are the first choice for the treatment of LDD. If conservative treatment for more than 6 weeks is ineffective or the effect is not obvious, surgical treatment needs to be considered. Conservative treatment promotes soft tissue healing, reduces spasm, and inhibits pain receptors through various physical therapies, while surgical treatment strives to completely resolve the compression and relieve neurological symptoms. In general, the two have their own advantages and disadvantages and complement each other. Therefore, the combination of the two is often used in clinical treatment for severe patients [21–23].

The mechanism of ESWT in the treatment of muscle spasm remains unclear. Previous studies have considered that it may directly act on muscle fibers to improve their stiffness and play an antispasmodic effect, while at the same time through a feedback mechanism to produce corresponding proteins in the cerebral cortex to inhibit pain [24]. Since the beginning of the use of shock waves to treat pain and muscle attachment diseases in the 1990s, more and more orthopedic doctors and rehabilitation technicians now advocate the use of shock waves to treat pain and mobility disorders caused by LDD, because shock wave therapy is not only a noninvasive treatment but also has been received good treatment feedback from patients.

In a study by Karolina et al. [25], 52 patients with LBP were enrolled. They reported that the pain improved significantly in the long-term follow-up, but these changes were not significant immediately. In addition, there was an increase in the functional state (ODI) after radial extracorporeal shock wave therapy compared with the basic assessment.

Behringer et al. [26] recruited 19 individuals affected by more than or equal to one calf cramp per week. The gastrocnemius muscle of the main affected leg was stimulated twice a week (intervention leg (IL)) (336 stimulation training, 30 Hz higher than the individual cramp threshold frequency). The another leg is viewed as a control (CL). Participants were informed to record all spontaneous muscle cramps from two weeks before the intervention to two weeks after the last NMES treatment. The results showed the number of spontaneous calf cramps in the two weeks after the intervention was lower. Not only that the remaining calf cramps in the legs tend to be less severe after the intervention. The applied stimulation program seems to provide an effective preventive strategy for patients who frequently experience calf cramps.

In a study by Lee et al. [27], 28 patients with low back pain (LBP) who were randomly assigned to two groups were recruited. The ESWT group consisted of 13 patients, and the control group consisted of 15 participants. There were no statistically significant differences in their age, height, and weight. All patients received a regular exercise rehabilitation program (30 minutes, twice a week, for 1.5 months) including stability training and core strength exercises. In the ESWT group, the patients received 2000 pulses each time, with a frequency of 5 Hz and a dose of 0.1 mJ/mm^2^ shock wave treatment. In the control group, the hyperthermia, ultrasound therapy, and transcutaneous electrical nerve stimulation (TENS) were performed every day. After the treatment, the VAS pain score of all patients showed that the pain was alleviated, but the ESWT treatment effect was better (*p* < 0.05).

So far, there is still not enough scientific standard studies to prove the clinical value of ESWT in the treatment of LDD. The results of previous research have made some progress, but further development is needed. The purpose of our study was to evaluate the effectiveness of ESWT in treating lower extremity muscle spasms in patients with LDD. From this study, ESWT seems to have a certain effect. Compared with traditional conservative treatment, the effectiveness seems to be better. Compared with surgical treatment, ESWT is more noninvasive, less harmful to patients, and more acceptable for treatment costs. However, most of the current research focuses on the effect of ESWT on poststroke spasm. In spinal surgery, more attention is paid to its pain relief. Therefore, the current efficacy of ESWT in the treatment of lower extremity muscle spasms caused by LDD is still lacking. Our research has initially proved the effectiveness of ESWT in the treatment of LDD. This RCT provides some suggestions for the use of ESWT in LDD clinical practice. We recommend that when using shock wave to treat LDD, keeping 2.5 bar, 5 Hz, 2000 times once, once every two days, and each course of treatment is 5 weeks.

## Figures and Tables

**Table 1:** Sociodemographic characteristics of patients.

	ESWT group (*n* = 78)	Control group (*n* = 44)	*z*	*p*
	Mean ± SD	Mean ± SD		
Age (year)	64.23 ± 10.68	61.31 ± 8.96	-1.126	0.26
Body mass index (kg/m^2^)	28.38 ± 2.47	27.10 ± 3.36	-1.884	0.06

ESWT: extracorporeal shock wave therapy; SD: standard deviation; Mann–Whitney *U* test.

**Table 2:** Inter- and intragroup comparisons of pain and Fugl-Meyer score (LL).

	ESWT group	Control group	*z*	*p*
	Mean ± SD	Mean ± SD		
Visual Analog Scale^1^	7.6 ± 1.1	7.2 ± 1.4	-0.129	0.76
Visual Analog Scale^2^	1.7 ± 0.3	2.3 ± 0.9	-4.589	<0.001^∗∗^‡
*z*	-5.379	-3.916		
*p*	<0.001^∗∗^†	<0.001^∗∗^†		
Lower limb (0-34)^1^	14.6 ± 2.7	16.5 ± 1.4	-1.153	0.24
Lower limb (0-34)^2^	31.3 ± 4.6	26.1 ± 2.3	-5.473	<0.001^∗∗^‡
*z*	-5.327	-2.582		
*p*	<0.001^∗∗^†	0.006†		
Number of cramps^1^ (times/hour)	5.7 ± 1.2	5.2 ± 2.3	-1.076	0.32
Number of cramps^2^ (times/hour)	1.3 ± 0.8	3.7 ± 1.4	-3.476	<0.001^∗∗^‡
*z*	-3.091	-2.036		
*p*	<0.001^∗∗^†	0.041^∗^		
Duration of cramps^1^ (second)	57.6 ± 8.7	63.4 ± 6.2	-0.586	0.592
Duration of cramps^2^ (second)	10.6 ± 5.3	21.8 ± 4.9	-4.277	<0.001^∗∗^‡
*z*	-4.736	-2.873		
*p*	<0.001^∗∗^†	-2.634		

ESWT: extracorporeal shock wave therapy; SD: standard deviation. ^1^Pretreatment. ^2^Posttreatment. ^∗^*p* < 0.05; ^∗∗^*p* < 0.001; †Wilcoxon test; ‡Mann–Whitney *U* test.

## Data Availability

The data used to support the findings of this study are currently under embargo while the research findings are commercialized. Requests for data (6/12 months), after publication of this article, will be considered by the corresponding author.
